# Impact of free-living pattern of sedentary behaviour on intra-day glucose regulation in type 2 diabetes

**DOI:** 10.1007/s00421-019-04261-z

**Published:** 2019-11-08

**Authors:** Aye C. Paing, Kathryn A. McMillan, Alison F. Kirk, Andrew Collier, Allan Hewitt, Sebastien F. M. Chastin

**Affiliations:** 1 School of Health and Life Sciences, Glasgow Caledonian University, Glasgow, UK; 2 Physical Activity for Health Group, School of Psychological Sciences and Health, University of Strathclyde, Glasgow, UK; 3 Department of Movement and Sports Science, Ghent University, Ghent, Belgium

**Keywords:** Type 2 diabetes, Glucose targets, Glycaemic control, Sedentary behaviour, Physical activity, Breaks in sedentary time

## Abstract

**Purpose:**

To investigate how the pattern of sedentary behaviour affects intra-day glucose regulation in type 2 diabetes.

**Methods:**

This intensive longitudinal study was conducted in 37 participants with type 2 diabetes (age, 62.8 ± 10.5 years). Glucose and sedentary behaviour/physical activity were assessed with a continuous glucose monitoring (Abbott FreeStyle Libre) and an activity monitor (activPAL3) for 14 days. Multiple regression models with generalised estimating equations (GEEs) approach were used to assess the associations of sedentary time and breaks in sedentary time with pre-breakfast glucose, pre-lunch glucose, pre-dinner glucose, post-breakfast glucose, post-lunch glucose, post-dinner glucose, bedtime glucose, the dawn phenomenon, time in target glucose range (TIR, glucose 3.9–10 mmol/L) and time above target glucose range (TAR, glucose > 10 mmol/L).

**Results:**

Sedentary time was associated with higher pre-breakfast glucose (*p* = 0.001), pre-dinner glucose (*p* < 0.001), post-lunch glucose (*p* = 0.005), post-dinner glucose (*p* = 0.013) and the dawn phenomenon (*p* < 0.001). Breaks in sedentary time were associated with lower pre-breakfast glucose (*p* = 0.023), pre-dinner glucose (*p* = 0.023), post-breakfast glucose (*p* < 0.001) and the dawn phenomenon (*p* = 0.004). The association between sedentary time and less TIR (*p* = 0.022) and the association between breaks in sedentary time and more TIR (*p* = 0.001) were also observed.

**Conclusions:**

Reducing sedentary time and promoting breaks in sedentary time could be clinically relevant to improve intra-day glucose regulation in type 2 diabetes.

**Electronic supplementary material:**

The online version of this article (10.1007/s00421-019-04261-z) contains supplementary material, which is available to authorized users.

## Introduction

Sedentary time (time spent sitting or reclining during waking hours) is highly prevalent in people with type 2 diabetes. People with type 2 diabetes spend approximately 64% of their waking hours in sedentary behaviour (van der Berg et al. 2016). There is cross-sectional evidence that sedentary time is associated with high fasting glucose, 2-h postprandial glucose after a test meal, insulin resistance and HbA1c (glycated haemoglobin) in people with type 2 diabetes (Healy et al. 2007; Helmerhorst et al. 2009; Sardinha et al. 2017). On the other hand, breaks in sedentary time, defined as short period of standing or walking in between two periods of sitting/reclining, have beneficial associations with fasting glucose, 2-h postprandial glucose after a test meal and insulin resistance in those with type 2 diabetes (Healy et al. 2008; Sardinha et al. 2017; Tremblay et al. 2017). These findings suggest that sedentary time and breaks in sedentary time may impact intra-day glucose regulation, such as pre-breakfast glucose (fasting glucose), pre-lunch glucose, pre-dinner glucose, post-breakfast glucose, post-lunch glucose, post-dinner glucose, bedtime glucose, the dawn phenomenon, time in target glucose range (TIR, glucose 3.9–10 mmol/L) and time above target glucose range (TAR, glucose > 10 mmol/L). If this is the case, then there is a possibility that reduction of sedentary time with activity breaks could be used as a potential therapeutic intervention to help regulate glycaemia. However, limited evidence is available to support the associations of sedentary time and breaks is sedentary time with pre-breakfast glucose, pre-lunch glucose, pre-dinner glucose, post-breakfast glucose, post-lunch glucose, post-dinner glucose, bedtime glucose, the dawn phenomenon, TIR and TAR in free-living settings. There is an evidence-based global guideline for type 2 diabetes produced by the International Diabetes Federation (IDF), and it suggests that pre-meal glucose and post-meal glucose control are crucial to achieve recommended HbA1c < 7% (53 mmol/mol) and to reduce diabetes-related complications (International Diabetes Federation Guideline Development Group 2014). Additionally, bedtime glucose, the dawn phenomenon and TIR have been shown to be associated with HbA1c (Monnier et al. 2013; Wei et al. 2014; Vigersky and McMahon 2018; Battelino et al. 2019). Therefore, glucose regulation throughout the day is important in those with type 2 diabetes. This study aimed to explore if sedentary time and breaks in sedentary are associated with pre-breakfast glucose, pre-lunch glucose, pre-dinner glucose, post-breakfast glucose, post-lunch glucose, post-dinner glucose, bedtime glucose, the dawn phenomenon, TIR and TAR in people with type 2 diabetes with habitual diet and physical activity patterns.

## Methods

### Study design

The present study was an intensive longitudinal study (Bolger and Laurenceau 2013). It was approved by the University Ethics Committee (UEC) of University of Strathclyde. The study was conducted in accordance with the Declaration of Helsinki, and written informed consent was obtained from all participants.

The study involved two short visits to the University laboratory or convenient location (e.g. participant’s home). During visit one, participants were fitted with a continuous glucose monitoring (CGM, Abbott FreeStyle Libre) and an activPAL3 activity monitor (PAL Technologies, Glasgow, UK). The researcher collected participants’ demographic data. Participants then returned home and completed their normal daily living for up to 14 days. To ensure compliance with protocol, participants were requested to maintain their habitual diet and physical activity patterns throughout the study. Participants were also provided with sleep diary and 24-h Dietary Recall Form to record their habitual bedtime, waking time, meal time, dietary intake and medication. At visit two, the CGM and activPAL3 were removed, and this was the end of study for each participant.

### Participants

Participants were recruited between February 2016 and February 2017 through advertising within the staff of two universities, Diabetes Balance magazine, the Diabetes UK website and diabetes support groups in the Glasgow community. Eligibility criteria included diagnosed type 2 diabetes and age ≥ 18 years. Exclusion criteria included pregnancy, alcohol and substance abuse, insulin therapy, age < 18 years, liver and renal diseases and cancer.

### Assessment of carbohydrate intake and demographic variables

Demographic variables such as age, gender, duration of diabetes, anti-diabetes medication, waist circumference and body mass index (BMI) were collected. Carbs & Cals Counter and 24-h Dietary Recall Form were used to calculate daily carbohydrate intake (g/day) for each participant (Cheyette and Balolia 2013).

### Assessment of sedentary time and breaks in sedentary time

The activPAL3 activity monitor was waterproofed and was then attached to the anterior aspect of the right thigh using hypoallergenic dressing. Participants were requested to wear the device continuously for up to 14 days, and they were also provided with extra hypoallergenic dressing and information on how to reattach the device if it has fallen off. Participants were also asked to record the time if they remove the device or the device has fallen off, and it was noted that all participants wore the device continuously for up to 14 days in this study. This device was validated and accurately monitors the start and duration of sitting, lying, standing and walking for 14 days (Lyden et al. 2012, 2017). This device also provides valid estimates of breaks in sedentary time (Lyden et al. 2012, 2017). The activPAL3™ software (version 7.2.32) was used to download the data from this device.

Sedentary time (h/day), sleeping time (h/day), walking time (h/day), moderate to vigorous physical activity (MVPA) time (min/day) and number of breaks in sedentary time (*n*/day) were calculated for each day, but the first and final days, which do not have full 24-h recording, were excluded. Sedentary time (h/day) was computed after sleeping time was excluded using the sleep diary and activPAL events file (Edwardson et al. 2016). Transitions from sitting or lying condition to standing or stepping condition during waking time were used to calculate the number of breaks in sedentary time. A cadence greater or equal to 100 steps/min was classified as MVPA (Marshall et al. 2009).

### Assessment of glucose profiles

The CGM used in this study records accurate interstitial glucose every 15 min for up to 14 days (Bailey et al. 2015). This device was worn on the back of the upper arm. Participants were provided with the reader to scan and retrieve glucose data at least every 8 h. FreeStyle Libre software (version 1.0) was used to download the glucose data.

Glucose values before the start of breakfast, lunch and dinner were defined as pre-breakfast glucose, pre-lunch glucose and pre-dinner glucose (Thomas et al. 2016). Glucose values during 2 h after breakfast, lunch and dinner were, respectively, used to calculate post-breakfast glucose, post-lunch glucose and post-dinner glucose (Thomas et al. 2016). Meal times recorded in 24-h Dietary Recall Form were used to determine breakfast, lunch and dinner times. The CGM glucose value at bedtime, which was confirmed by the sleep diary and activPAL events file, was defined as bedtime glucose. The dawn phenomenon was defined as an increase in the CGM glucose value from the nocturnal nadir glucose to pre-breakfast glucose (Monnier et al. 2013). It was considered to be absent and was recorded as zero when pre-breakfast glucose was lower than all nocturnal glucose values (Monnier et al. 2012). After excluding the first and final days with less than 24-h data, 10614 glucose measurements were included to calculate pre-breakfast glucose, pre-lunch glucose, pre-dinner glucose, post-breakfast glucose, post-lunch glucose, post-dinner glucose, bedtime glucose and the dawn phenomenon for each day. Across all participants, 4.2% of data points for these glucose profiles (451 of 10614 glucose measurements) were missing, and within-individual mean substitution was applied to deal with missing data points (Cheema 2014).

Daily TIR (glucose 3.9–10 mmol/L) and TAR (glucose > 10 mmol/L) were also calculated for each participant (Battelino et al. 2019). Each missing glucose data point represents 15 min missing data time, and daily TIR and TAR were calculated as % of recording h/day using normalisation method (e.g. TIR = [time in glucose range of 3.9–10 mmol/L/(24 h—daily missing data time)] × 100) (Vigersky and McMahon 2018; Battelino et al. 2019). Participants reported HbA1c from their last visits to general practitioner, diabetes specialist nurse and diabetes clinic.

### Statistical analyses

To be included in the final analysis, participants were required to have minimum 3 days of concurrent and continuous glucose and activity data. Multiple regression models with generalised estimating equations (GEEs) approach were used to examine the associations of sedentary time and breaks in sedentary time with pre-breakfast glucose, pre-lunch glucose, pre-dinner glucose, post-breakfast glucose, post-lunch glucose, post-dinner glucose, bedtime glucose, the dawn phenomenon, TIR and TAR. The GEE approach allowed us to analyse continuous and longitudinal data while accounting for intra-individual correlations (Windt et al. 2018). All dependent variables, except for TIR, TAR and pre-lunch glucose, were normally distributed. Regression models for normally distributed variables were fitted under the linearity assumption. TIR, TAR and pre-lunch glucose were positively skewed, and regression models for these variables were fitted under the assumption of a gamma distribution (Manne et al. 2011). Model 1 was adjusted for age, gender, sleeping time, walking time and carbohydrate intake. Model 2 was adjusted for variables in Model 1 and BMI and duration of diabetes. Model 1 and Model 2 investigating the associations between breaks in sedentary time and glucose variables were also adjusted for sedentary time. The quasi-likelihood under the independence model criterion (QIC) method was used to identify the best working correlation matrix, which has the smallest QIC value (Pan 2001; Cui and Qian 2007). In this study, the independent correlation matrix showed the smallest QIC value, and the regression analyses were therefore conducted under the assumption of an independent correlation matrix. To determine the presence of multicollinearity between independent variables, thresholds for correlation coefficient > 0.7 and Variance Inflation Factor > 10 were used (Dormann et al. 2013). There was no evidence of multicollinearity in the regression models (Correlation coefficients < 0.5, Variance Inflation Factors < 2). The results are reported as unstandardised regression coefficient (B) with 95% confidence interval (CI) and mean with standard deviation (SD) unless otherwise indicated. Significance level was set at *p* value ≤ 0.05. Data were prepared using Microsoft Excel 2016, and all data analyses were conducted with IBM SPSS Statistics software (version 24.0).

A sensitivity analysis was conducted to investigate whether the associations of sedentary time and breaks in sedentary time with glucose variables were affected by adjusting for MVPA time rather than walking time in Model 1 and Model 2.

## Results

Table 1 describes characteristics of participants. Thirty-seven participants (age, 62.8 ± 10.5 years; BMI, 29.6 ± 6.8 kg/m^2^) (mean ± SD) with 10 ± 3.4 days (mean ± SD) of the CGM and activPAL3 recording time were included in final analyses.

**Table 1:** Characteristics of participants

Characteristics of participants	
Number of participants (men/women) (*n*)	37 (14/23)
Age (years)	62.8 ± 10.5
BMI (kg/m^2^)	29.6 ± 6.8
Waist circumference (cm)	99.8 ± 11.9
HbA1c (%), (*n* = 15 missing)	6.6 ± 0.9
HbA1c (mmol/mol), (*n* = 15 missing)	47.7 ± 10.6
Duration of diabetes (years)	5.9 ± 4.7
Diabetes management (*n*)	
No medication/diet modification alone	12
Metformin	18
Metformin + sulphonylurea	5
Metformin + gliptin	1
Metformin + sulphonylurea + gliptin	1
Carbohydrate intake (g/day)	125.3 ± 21.1
Sedentary time (h/day)	9.8 ± 1.8
Breaks in sedentary time (*n*/day)	52 ± 13
MVPA time (min/day)	32.1 ± 22.7
Walking time (h/day)	1.6 ± 0.7
Sleeping time (h/day)	8.3 ± 1.4
Pre-breakfast glucose (mmol/L)	6.7 ± 1.8
Pre-lunch glucose (mmol/L)	6.5 ± 1.7
Pre-dinner glucose (mmol/L)	6.7 ± 1.6
Post-breakfast glucose (mmol/L)	8.5 ± 1.9
Post-lunch glucose (mmol/L)	7.5 ± 1.6
Post-dinner glucose (mmol/L)	7.9 ± 1.7
Bedtime glucose (mmol/L)	7.1 ± 1.7
The dawn phenomenon (mmol/L)	1.6 ± 0.6
TIR (% of recording h/day)	84.9 ± 16.8
TAR (% of recording h/day)	11.4 ± 16.6

Data are mean ± SD or number (*n*)

*BMI* body mass index, *MVPA* moderate to vigorous physical activity

Table 2 shows the results of the regression models investigating the associations between sedentary time and glucose variables. In Model 1, sedentary time was significantly associated with higher pre-breakfast glucose (0.18 mmol/L/h, 95% CI 0.07; 0.28), pre-dinner glucose (0.21 mmol/L/h, 95% CI 0.11; 0.32), post-lunch glucose (0.15 mmol/L/h, 95% CI 0.04; 0.26), post-dinner glucose (0.10 mmol/L/h, 95% CI 0.02; 0.17) and the dawn phenomenon (0.16 mmol/L/h, 95% CI 0.11; 0.21). A significant association between sedentary time and less TIR (− 1.15% of recording h/day/h, 95% CI − 2.13; − 0.16) was also found in Model 1. In Model 2, the associations of sedentary time with higher pre-breakfast glucose (0.19 mmol/L/h, 95% CI 0.07; 0.29), pre-dinner glucose (0.17 mmol/L/h, 95% CI 0.07; 0.28), post-lunch glucose (0.17 mmol/L/h, 95% CI 0.06; 0.28), post-dinner glucose (0.15 mmol/L/h, 95% CI 0.04; 0.26) and the dawn phenomenon (0.06 mmol/L/h, 95% CI 0.00; 0.12) and less TIR (− 1.09% of recording h/day/h, 95% CI − 2.07; − 0.11) remained significant. However, sedentary time was not significantly associated with pre-lunch glucose, post-breakfast glucose, bedtime glucose and TAR in both Model 1 and Model 2.

**Table 2:** Associations between sedentary time and glucose variables

Glucose variables	Number of observations (*n*)	B (95% CI)	*P* value
Model 1			
Pre-breakfast glucose (mmol/L)	366	0.18 (0.07, 0.28)	0.001
Pre-lunch glucose (mmol/L)	366	0.01 (− 0.11, 0.12)	0.884
Pre-dinner glucose (mmol/L)	366	0.21 (0.11, 0.32)	<0.001
Post-breakfast glucose (mmol/L)	366	0.09 (− 0.03, 0.21)	0.127
Post-lunch glucose (mmol/L)	366	0.15 (0.04, 0.26)	0.005
Post-dinner glucose (mmol/L)	366	0.10 (0.02, 0.17)	0.013
Bedtime glucose (mmol/L)	366	0.07 (− 0.05, 0.19)	0.260
The dawn phenomenon (mmol/L)	366	0.16 (0.11, 0.21)	<0.001
TIR (% of recording h/day)	366	− 1.15 (− 2.13, − 0.16)	0.022
TAR (% of recording h/day)	245	0.81 (− 0.32, 1.94)	0.164
Model 2			
Pre-breakfast glucose (mmol/L)	366	0.19 (0.07, 0.29)	0.001
Pre-lunch glucose (mmol/L)	366	− 0.02 (− 0.13, 0.09)	0.732
Pre-dinner glucose (mmol/L)	366	0.17 (0.07, 0.28)	0.001
Post-breakfast glucose (mmol/L)	366	0.10 (− 0.02, 0.22)	0.109
Post-lunch glucose (mmol/L)	366	0.17 (0.06, 0.28)	0.002
Post-dinner glucose (mmol/L)	366	0.15 (0.04, 0.26)	0.009
Bedtime glucose (mmol/L)	366	0.07 (− 0.05, 0.19)	0.266
The dawn phenomenon (mmol/L)	366	0.06 (0.00, 0.12)	0.037
TIR (% of recording h/day)	366	− 1.09 (− 2.07, − 0.11)	0.029
TAR (% of recording h/day)	245	0.42 (− 0.49, 1.34)	0.366

Data are presented as unstandardised regression coefficient (B) with 95% confidence interval (CI)

In the GEE models, B indicates the strength of the association and how much of the dependent variable is explained by the independent variable

Model 1 was adjusted for age, gender, sleeping time, walking time and carbohydrate intake

Model 2 was adjusted for variables in Model 1 and body mass index and duration of diabetes

The results of the regression models investigating the associations between breaks in sedentary time and glucose variables are reported in Table 3. Model 1 showed significant associations of breaks in sedentary time with lower pre-breakfast glucose (− 0.01 mmol/L/break, 95% CI − 0.02; − 0.001), pre-dinner glucose (− 0.02 mmol/L/break, 95% CI − 0.03; − 0.002), post-breakfast glucose (− 0.01 mmol/L/break, 95% CI − 0.02; − 0.01) and the dawn phenomenon (− 0.01 mmol/L/break, 95% CI − 0.01; − 0.002). Breaks in sedentary time were also associated with more TIR (0.18% of recording h/day/break, 95% CI 0.07; 0.29), but no significant associations of breaks in sedentary time with pre-lunch glucose, post-lunch glucose, post-dinner glucose, bedtime glucose and TAR were found in Model 1. In Model 2, a significant association of breaks in sedentary time with lower pre-breakfast glucose (− 0.01 mmol/L/break, 95% CI − 0.01; − 0.003) was observed; however, the remaining glucose variables were not significantly associated with breaks in sedentary.

**Table 3:** Associations between breaks in sedentary time and glucose variables

Glucose variables	Number of observations (*n*)	B (95% CI)	*p* value
Model 1			
Pre-breakfast glucose (mmol/L)	366	− 0.01 (− 0.02, − 0.001)	0.023
Pre-lunch glucose (mmol/L)	366	− 0.01 (− 0.02, 0.01)	0.378
Pre-dinner glucose (mmol/L)	366	− 0.02 (− 0.03, − 0.002)	0.023
Post-breakfast glucose (mmol/L)	366	− 0.01 (− 0.02, − 0.01)	< 0.001
Post-lunch glucose (mmol/L)	366	− 0.003 (− 0.01, 0.01)	0.537
Post-dinner glucose (mmol/L)	366	− 0.01 (− 0.02, 0.01)	0.329
Bedtime glucose (mmol/L)	366	− 0.01 (− 0.02, 0.01)	0.254
The dawn phenomenon (mmol/L)	366	− 0.01 (− 0.01, − 0.002)	0.004
TIR (% of recording h/day)	366	0.18 (0.07, 0.29)	0.001
TAR (% of recording h/day)	245	− 0.08 (− 0.18, 0.02)	0.129
Model 2			
Pre-breakfast glucose (mmol/L)	366	− 0.01 (− 0.01, − 0.003)	0.002
Pre-lunch glucose (mmol/L)	366	0.01 (− 0.01, 0.02)	0.356
Pre-dinner glucose (mmol/L)	366	− 0.003 (− 0.02, 0.01)	0.616
Post-breakfast glucose (mmol/L)	366	0.01 (− 0.004, 0.02)	0.154
Post-lunch glucose (mmol/L)	366	0.01 (− 0.004, 0.02)	0.207
Post-dinner glucose (mmol/L)	366	0.004 (− 0.01, 0.02)	0.555
Bedtime glucose (mmol/L)	366	0.004 (− 0.01, 0.02)	0.523
The dawn phenomenon (mmol/L)	366	− 0.003 (− 0.01, 0.004)	0.391
TIR (% of recording h/day)	366	0.08 (− 0.03, 0.19)	0.137
TAR (% of recording h/day)	245	0.05 (− 0.03, 0.14)	0.230

Data are presented as unstandardised regression coefficient (B) with 95% confidence interval (CI)

In the GEE models, B indicates the strength of the association and how much of the dependent variable is explained by the independent variable

Model 1 was adjusted for age, gender, sleeping time, walking time, carbohydrate intake and sedentary time

Model 2 was adjusted for variables in Model 1 and body mass index and duration of diabetes

A sensitivity analysis showed that the associations of sedentary time and breaks in sedentary time with glucose variables were independent of MVPA time. Sedentary time was associated with higher pre-breakfast glucose, post-lunch glucose, the dawn phenomenon and TAR and lower TIR in Model 1, and the associations with higher pre-breakfast glucose and post-lunch glucose and lower TIR remained significant in Model 2 (Supplemental Table 1). Breaks in sedentary time were associated with lower pre-dinner glucose and higher TIR in Model 1 and lower pre-breakfast glucose in Model 2 (Supplemental Table 2).

## Discussion

This study investigated, for the first time in people with type 2 diabetes, the impact of the pattern of sedentary behaviour on intra-day glycaemic control in free-living conditions using objective measurement methods. This study shows that results obtained in the laboratory transfer to free-living conditions (Chastin et al. 2015). Experimental studies showed that breaking sedentary time impacts glycaemic control, but these studies compared very extreme conditions (continuous sitting for 5–9 h vs. breaking sitting every 20 min or 30 min) and were not conducted in ecologically valid settings (Chastin et al. 2015). The use of continuous monitoring and intensive longitudinal methods enables to show that sedentary behaviour does change glycaemic control during the day in free-living conditions. This suggests that behavioural intervention aimed at modifying sedentary behaviour in type 2 diabetes could be used as a therapeutic modality to control glycaemia throughout the day.

There is evidence that pre-breakfast glucose, pre-dinner glucose, post-breakfast glucose and post-lunch glucose are associated with cardiovascular complications and all-cause mortality in those with type 2 diabetes (Cavalot et al. 2011; Tanaka 2012; Jiang et al. 2017). This highlights the importance of glucose control before and after meals, and every 1 mmol/L increase in pre-meal glucose and post-meal glucose can increase the risk of a cardiovascular event by 11% and 8%, respectively (Kilpatrick et al. 2008). However, it has been shown that glucose control before and after meals, particularly pre-breakfast glucose and post-breakfast glucose, are suboptimal in people with type 2 diabetes, even in those taking anti-diabetes agents (van Dijk et al. 2011; Paing et al. 2018a). Therefore, high incidence of diabetes-related complications tends to occur in people with type 2 diabetes (Nazimek-Siewniak et al. 2002; Cavalot et al. 2011; Jelinek et al. 2017). Considering the fact that type 2 diabetes is a heterogeneous condition, modifiable underlying factors should also be addressed, in addition to anti-diabetes agents, to improve clinical outcomes (Hartz et al. 2006). This study suggests that 4.8-h, 5.6-h, 6.7-h and 10-h decrease in sedentary time may produce a clinically meaningful reduction (1 mmol/L) in pre-dinner glucose, pre-breakfast glucose, post-lunch glucose and post-dinner glucose, respectively. The detrimental associations of sedentary time with pre-breakfast glucose, pre-dinner glucose, post-lunch glucose and post-dinner glucose observed in this study support the findings of previous cross-sectional studies, which reported the associations of sedentary time with high fasting glucose and postprandial glucose after a test meal (Healy et al. 2007; Sardinha et al. 2017). In addition, this study suggests that 50 breaks in sedentary time may translate to 1 mmol/L decrease in pre-dinner glucose, and 100 breaks in sedentary time may translate to 1 mmol/L decrease in pre-breakfast glucose and post-breakfast glucose. Therefore, reducing sedentary time with frequent activity breaks could be an important modifiable factor to improve glucose control before and after meals and clinical outcomes in type 2 diabetes.

This study provides initial confirmation of the associations of sedentary time and breaks in sedentary time with the dawn phenomenon in free-living settings. It showed that sedentary time can be a predictor of an increase in the dawn phenomenon and breaks in sedentary time can be predictive of a reduction in the dawn phenomenon. This finding is congruent with previous experimental evidence (Paing et al. 2019). Previous studies found that oral anti-diabetes agents cannot produce adequate control of the dawn phenomenon in people with type 2 diabetes (Monnier et al. 2007, 2012, 2013). Our findings suggest that each 7-h decrease in sedentary time and 111 breaks in sedentary time may result in the reduction of the dawn phenomenon by > 1.1 mmol/L, which is a clinically validated threshold for the dawn phenomenon (Monnier et al. 2013), and thus may produce a clinically meaningful improvement in control of the dawn phenomenon. Because, the magnitude of the dawn phenomenon > 1.1 mmol/L corresponds to an increase in HbA1c of 0.4% (4 mmol/mol) (Monnier et al. 2013), and even a 1% decrease in HbA1c is associated with 37% decrease in microvascular complications, 14% decrease in myocardial infarction and 21% decrease in diabetes-related mortality (Stratton et al. 2000).

The use of time spent within the glucose range of 3.9–10 mmol/L as a threshold for TIR has been clinically validated, and a recent consensus on the CGM glucose measurements developed by an international group of experts has recommended TIR as a key metric of glucose regulation (Battelino et al. 2019). An increase in TIR of 10% can lead to a decrease in HbA1c of about 0.5% (5 mmol/mol) (Battelino et al. 2019; Beck et al. 2019a), and the prevalence of diabetes-related complications is inversely associated with TIR in type 2 diabetes (Lu et al. 2018; Beck et al. 2019b). A previous study showed that each 10% reduction in TIR increases the risk of microalbuminuria development by 40% and retinopathy progression by 64% (Beck et al. 2019b). An important point of the present study is that it is the first to use this clinically validated TIR as an outcome variable, and it demonstrates the association between sedentary time and less TIR and the association between breaks in sedentary time and more TIR. This evidence extends the observation of previous studies (Fritschi et al. 2016; Paing et al. 2018b), and suggests that 8.7-h decrease in sedentary time and 56 breaks in sedentary time may translate to a clinically meaningful increase in TIR of 10%. We suggest that there may be a causal link between sedentary patterns and TIR, and reducing sedentary time with frequent activity breaks combined with anti-diabetes agents may produce more prominent clinical effect than anti-diabetes agents alone. This remains to be explored in definitive randomised controlled trials.

The present study has several strengths. First, intensive longitudinal design was used, and objective measurements of sedentary time and breaks in sedentary time were assessed with the activPAL3, following the recommended guidelines such as using 24 h data and the activPAL events file for data processing, providing sleep diary and being transparent about activity data processing (Edwardson et al. 2016; Dall et al. 2018). Second, the CGM used in this study provided accurate interstitial glucose data for up to 14 days and allowed us to examine daily glucose profiles throughout the study period (Bailey et al. 2015). The assessment of the dawn phenomenon was only possible with the use of CGM in this study (Monnier et al. 2013). Finally, potential confounders such as age, gender, sleeping time, walking time, carbohydrate intake, BMI and duration of diabetes, which might influence glucose control, were adjusted in statistical analyses (Morselli et al. 2010; Morgan et al. 2012; DiPietro et al. 2013; Hajian-Tilaki and Heidari 2015; Kautzky-Willer et al. 2015; Shamshirgaran et al. 2017).

This study also has some limitations, which could be addressed in future studies. A small sample size was used, which is often the case in intensive longitudinal studies and studies using the CGM due to cost and burden. Therefore, the present study might be underpowered to observe the associations of sedentary time and breaks in sedentary time with some glucose profiles. Moreover, participants with diet modification alone or metformin ± sulphonylurea ± gliptin were included in this study, and the sample with different anti-diabetes agents should be considered in future studies to establish generalisability of results. Furthermore, whether the sample meet guidelines for MVPA was not firmly confirmed in this study because at least 7 days of activity data are recommended to interpret physical activity level, and approximately 27% of our participants (data not shown) reported only 3–6 days of activity data (Edwardson et al. 2016). In addition, participants’ habitual diet, physical activity and sedentary patterns might be influenced by wearable devices such as the CGM and activPAL3 in this study. Finally, the associations between sedentary time and breaks in sedentary time and glucose control were cross-sectional, and further experimental studies are required to confirm cause–effect relationship.

In conclusion, the present study suggests that better intra-day glycaemic control could be attained by reduction of sedentary time and promoting breaks in sedentary time in daily clinical practice. Further experimental studies manipulating sedentary behaviour in free-living conditions are required to fully confirm that causal link and understand how manipulating sedentary time could be used as a therapeutic modality.

## Electronic supplementary material

Below is the link to the electronic supplementary material.

Supplementary material 1 (DOCX 14 kb)

Supplementary material 2 (DOCX 14 kb)

## Abbreviations

BMI: Body mass index
CGM: Continuous glucose monitoring
GEEs: Generalised estimating equations
HbA1c: Glycated haemoglobin
IDF: International Diabetes Federation
MVPA: Moderate to vigorous physical activity
QIC: Quasi-likelihood under the independence model criterion
TIR: Time in target glucose range
TAR: Time above target glucose range
