# Supplementary material for: Impact of free-living pattern of sedentary behaviour on intra-day glucose regulation in type 2 diabetes
Source: Eur J Appl Physiol. 2019 Nov 8;120(1):171–9. doi: 10.1007/s00421-019-04261-z (PMC6969863; doi:10.1007/s00421-019-04261-z)
Supplement: Supplementary file 1 — Supplementary material 1 (DOCX 14 kb) [file 421_2019_4261_MOESM1_ESM.docx]

**Supplemental Table 1** Associations between sedentary time and glucose variables

| Glucose variables | Number of observations (*n*) | B (95% CI) | *p* value |
| --- | --- | --- | --- |
| Model 1 |  |  |  |
| Pre-breakfast glucose (mmol/L) | 366 | 0.12 (0.03, 0.21) | 0.012 |
| Pre-lunch glucose (mmol/L) | 366 | -0.02 (-0.12, 0.08) | 0.684 |
| Pre-dinner glucose (mmol/L) | 366 | 0.04 (-0.07, 0.14) | 0.500 |
| Post-breakfast glucose (mmol/L) | 366 | 0.002 (-0.11, 0.11) | 0.976 |
| Post-lunch glucose (mmol/L) | 366 | 0.12 (0.02, 0.21) | 0.014 |
| Post-dinner glucose (mmol/L) | 366 | 0.05 (-0.05, 0.15) | 0.307 |
| Bedtime glucose (mmol/L) | 366 | 0.08 (-0.03, 0.19) | 0.140 |
| The dawn phenomenon (mmol/L) | 366 | 0.06 (0.01, 0.11) | 0.015 |
| TIR (% of recording h/day) | 366 | -1.36 (-2.22, -0.49) | 0.002 |
| TAR (% of recording h/day) | 245 | 1.13 (0.17, 2.08) | 0.021 |
| Model 2 |  |  |  |
| Pre-breakfast glucose (mmol/L) | 366 | 0.13 (0.04, 0.22) | 0.005 |
| Pre-lunch glucose (mmol/L) | 366 | -0.05 (-0.15, 0.05) | 0.312 |
| Pre-dinner glucose (mmol/L) | 366 | 0.06 (-0.04, 0.16) | 0.223 |
| Post-breakfast glucose (mmol/L) | 366 | 0.01 (-0.09, 0.12) | 0.833 |
| Post-lunch glucose (mmol/L) | 366 | 0.14 (0.05, 0.24) | 0.003 |
| Post-dinner glucose (mmol/L) | 366 | 0.08 (-0.01, 0.18) | 0.095 |
| Bedtime glucose (mmol/L) | 366 | 0.09 (-0.02, 0.19) | 0.116 |
| The dawn phenomenon (mmol/L) | 366 | 0.05 (-0.01, 0.09) | 0.087 |
| TIR (% of recording h/day) | 366 | -1.27 (-2.13, -0.41) | 0.004 |
| TAR (% of recording h/day) | 245 | 0.57 (-0.23, 1.37) | 0.163 |

Data are presented as unstandardised regression coefficient (B) with 95% confidence interval (CI).

In the GEE models, B indicates the strength of the association and how much of the dependent variable is explained by the independent variable.

Model 1 was adjusted for age, gender, sleeping time, moderate to vigorous physical activity time and carbohydrate intake. Model 2 was adjusted for variables in Model 1 and body mass index and duration of diabetes.
