# Supplementary material for: Impact of free-living pattern of sedentary behaviour on intra-day glucose regulation in type 2 diabetes
Source: Eur J Appl Physiol. 2019 Nov 8;120(1):171–9. doi: 10.1007/s00421-019-04261-z (PMC6969863; doi:10.1007/s00421-019-04261-z)
Supplement: Supplementary file 2 — Supplementary material 2 (DOCX 14 kb) [file 421_2019_4261_MOESM2_ESM.docx]

**Supplemental Table 2** Associations between breaks in sedentary time and glucose variables

| Glucose variables | Number of  observations (*n*) | B (95% CI) | *p* value |
| --- | --- | --- | --- |
| Model 1 |  |  |  |
| Pre-breakfast glucose (mmol/L) | 366 | -0.07 (-0.01, 0.01) | 0.990 |
| Pre-lunch glucose (mmol/L) | 366 | -0.003 (-0.02, 0.01) | 0.568 |
| Pre-dinner glucose (mmol/L) | 366 | -0.01 (-0.03, 0.00) | 0.044 |
| Post-breakfast glucose (mmol/L) | 366 | 0.004 (-0.01, 0.02) | 0.542 |
| Post-lunch glucose (mmol/L) | 366 | 0.003 (-0.01, 0.02) | 0.561 |
| Post-dinner glucose (mmol/L) | 366 | -0.003 (-0.02, 0.01) | 0.586 |
| Bedtime glucose (mmol/L) | 366 | -0.01 (-0.02, 0.01) | 0.262 |
| The dawn phenomenon (mmol/L) | 366 | -0.004 (-0.01, 0.002) | 0.228 |
| TIR (% of recording h/day) | 366 | 0.19 (0.08, 0.29) | 0.001 |
| TAR (% of recording h/day) | 245 | -0.07 (-0.17, 0.02) | 0.131 |
| Model 2 |  |  |  |
| Pre-breakfast glucose (mmol/L) | 366 | -0.02 (-0.02, -0.01) | <0.001 |
| Pre-lunch glucose (mmol/L) | 366 | 0.01 (-0.004, 0.02) | 0.190 |
| Pre-dinner glucose (mmol/L) | 366 | 0.00 (-0.01, 0.01) | 0.973 |
| Post-breakfast glucose (mmol/L) | 366 | 0.01 (-0.003, 0.02) | 0.217 |
| Post-lunch glucose (mmol/L) | 366 | 0.01 (0.00, 0.02) | 0.058 |
| Post-dinner glucose (mmol/L) | 366 | 0.01 (-0.004, 0.02) | 0.214 |
| Bedtime glucose (mmol/L) | 366 | 0.01 (-0.01, 0.02) | 0.437 |
| The dawn phenomenon (mmol/L) | 366 | -0.002 (-0.01, 0.004) | 0.579 |
| TIR (% of recording h/day) | 366 | 0.08 (-0.03, 0.19) | 0.147 |
| TAR (% of recording h/day) | 245 | 0.03 (-0.04, 0.11) | 0.394 |

Data are presented as unstandardised regression coefficient (B) with 95% confidence interval (CI).

In the GEE models, B indicates the strength of the association and how much of the dependent variable is explained by the independent variable.

Model 1 was adjusted for age, gender, sleeping time, moderate to vigorous physical activity time, carbohydrate intake and sedentary time. Model 2 was adjusted for variables in Model 1 and body mass index and duration of diabetes.
